# Probing the Druggablility on the Interface of the Protein–Protein Interaction and Its Allosteric Regulation Mechanism on the Drug Screening for the CXCR4 Homodimer

**DOI:** 10.3389/fphar.2019.01310

**Published:** 2019-11-07

**Authors:** Liting Shen, Yuan Yuan, Yanzhi Guo, Menglong Li, Chuan Li, Xuemei Pu

**Affiliations:** ^1^College of Chemistry, Sichuan University, Chengdu, China; ^2^College of Management, Southwest University for Nationalities, Chengdu, China; ^3^College of Computer Science, Sichuan University, Chengdu, China

**Keywords:** target, GPCR dimer interface, druggability, regulation mechanism, computation

## Abstract

Modulating protein–protein interactions (PPIs) with small drug-like molecules targeting it exhibits great promise in modern drug discovery. G protein-coupled receptors (GPCRs) are the largest family of targeted proteins and could form dimers in living biological cells through PPIs. However, compared to drug development of the orthosteric site, there has been lack of investigations on the druggability of the PPI interface for GPCRs and its functional implication on experiments. Thus, in order to address these issues, we constructed a novel computational strategy, which involved in molecular dynamics simulation, virtual screening and protein structure network (PSN), to study one representative GPCR homodimer (CXCR4). One druggable pocket was identified in the PPI interface and one small molecule targeting it was screened, which could strengthen PPI mainly through hydrophobic interaction between the benzene rings of the PPI molecule and TM4 of the receptor. The PSN results further reveals that the PPI molecule could increase the number of the allosteric regulation pathways between the druggable pocket of the dimer interface to the orthostatic site for the subunit A but only play minor role for the other subunit B, leading to the asymmetric change in the volume of the binding pockets for the two subunits (increase for the subunit A and minor change for the subunit B). Consequently, the screening performance of the subunit A to the antagonists is enhanced while the subunit B is unchanged nearly, implying that the PPI molecule may be beneficial to enhance the drug efficacies of the antagonists. In addition, one main regulation pathway with the highest frequency was identified for the subunit A, which consists of Trp195^5.34^–Tyr190^ECL2^–Val196^5.35^–Gln200^5.39^–Asp262^6.58^–Cys28^N-term^, revealing their importance in the allosteric regulation from the PPI molecule. The observations from the work could provide valuable information for the development of the PPI drug-like molecule for GPCRs.

## Introduction

In living cells, only a few proteins perform their biological functions independently, and the vast majority (more than 80%) of proteins function through interacting with other molecules (Keskin et al., 2016; Wang et al., 2018b). It is estimated that there are approximately 130,000 to 650,000 protein–protein interactions (PPIs) in the human interactome (Venkatesan et al., 2009; Sheng et al., 2015; Tortorella et al., 2016), and targeting protein–protein interactions (PPIs) with small drug-like molecules (Sheng et al., 2015; Shin et al., 2017; Han et al., 2018) become one of the most promising methods in modern drug discovery (Li et al., 2017; Tang et al., 2019a; Tang et al., 2019b). If drugs could strengthen the PPI interaction or damage it, the function of PPI will be inevitably influenced. With increasing understanding of PPIs, significant progress has been made for investigations on PPI small drug-like molecules (Wells and McClendon, 2007; Jubb et al., 2012; Song et al., 2016; Shin et al., 2017). It was observed that the PPI molecules commonly have larger molecular weight, higher hydrophobicity, and lower solubility than most of traditional drug molecules (Villoutreix et al., 2012; Wang et al., 2018b). In previous studies, most of the drugs bind a targeted protein and inhibit it to form functional complexes with its binding partners, in turn influencing the downstream signals. For example, small molecule LEDGINs could block the interaction between HIV-1 integrase and human LEDGF/p75 so that it could inhibit HIV replication(Reddy et al., 2014). The peptidemimics MAIT was found to inhibit the migration of colorectal cells by disrupting APC–Asef interaction (Jiang et al., 2017). Although researches on strengthening PPI interaction are very limited with respect to inhibiting one, it is also highly valuable for some specific proteins. For example, ISD could strengthen the interaction between Neph1 and ZO-1 so that it could prevent podocyte injury and preserve glomerular filtration function (Sagar et al., 2017).

G protein-coupled receptors (GPCRs) are the largest membrane protein families with more than 800 members, which play key roles in various signal transductions. Approximate 50 percent of drugs target them (Rosenbaum et al., 2009; Venkatakrishnan et al., 2013; Lao et al., 2017). Monomers have long been recognized as functional units of GPCR signaling (Whorton et al., 2007; Maurice et al., 2011). However, recently increasing biochemical and biophysical evidences have indicated that the GPCR dimers and oligomers also exist in living biological cells (Ferré et al., 2014; Navarro et al., 2018; Pediani et al., 2018), which could significantly affect the signal transduction process of GPCRs like receptor activation, internalization, ligand binding and coupling with G protein (Huang et al., 2013; Xue et al., 2015; Damian et al., 2018). Some experimental works already found that positive or negative cooperativity exists between the two subunits of the GPCR dimer (Maurice et al., 2011). For example, when the ligand binds to one of the subunits, it will increase or decrease the binding affinity of another subunit to the ligand (Gherbi et al., 2015; Liu et al., 2017). Therefore, the GPCR dimers possess unique pharmacological profiles, being potential drug targets for the discovery of novel drugs.

Chemokine receptors are members of family A GPCRs, which regulate cell migration in development, immune system function and inflammatory diseases, thus being important therapeutic targets (Kufareva et al., 2014; Van Hout et al., 2018). CXCR4 is one of 23 known human chemokine receptors, which plays a key role in leukocyte trafficking, hematopoiesis, organ development and cancer metastases (Zweemer et al., 2014). It was revealed that CXCR4 is associated with more than 23 types of cancers (Wu et al., 2010; Nguyen et al., 2018). CXCR4 and related CC chemokine receptor 5 (CCR5) are not only the key regulators of signal transduction, but also involve in the entry of HIV-1 virus as coreceptors of HIV-1 into leukocytes (Shaik et al., 2019). Several observations suggested that the dimer may be the minimal functional units of the chemokine receptors and CXCR4 was demonstrated to form homo- or hetero-dimers (Percherancier et al., 2005; Muñoz et al., 2012). In 2010, the crystal structure of the CXCR4 homo-dimer (PDBID: 3ODU) was resolved (Wu et al., 2010). Chemotaxis assay shows that the migration index of T-REx-293 cells stably transfected with CXCR4 gene changes with the oligomeric status of CXCR4, indicating a correlation between the functions and the oligomeric status of CXCR4 (Lao et al., 2017). These findings clearly indicate that the polymerization of GPCRs could affect the structure and the function of the receptors. Therefore, it is also valuable to design small drug-like molecules targeting PPIs of GPCRs, which are beneficial to their therapeutic effects, to enhance their polymerization. However, the investigations on drug-like small molecules targeting the interface to enhance PPIs have been lacked so far. Thus, many questions have been remained to be unclear. For example, is the PPI interface druggable for GPCRs like CXCR4? What drugs could target the interface? How does the drug regulate the dimerization and the structure of the receptor, in turn influence its drug efficacy? In fact, these questions mainly involve in microscopic structure changes of the receptor upon the ligand bound the PPI interface. Therefore, it is highly desired to introduce computational techniques to assist the experiments to probe these issues.

Molecular dynamics (MD) simulation could acquire the structural evolution of proteins at the atomic level. Therefore, it become a powerful tool to study the structural and functional mechanisms for biological systems (Xue et al., 2018), including GPCRs. However, previous MD researches on GPCRs were mainly focused on the GPCR monomers, including their structures (Liang et al., 2017; Zhang et al., 2018a), interactions with ligands (Bai et al., 2014; Sader et al., 2018), activation mechanisms (Miao et al., 2015; Stanley et al., 2016), water channels (Yuan et al., 2013; Yuan et al., 2015) and so on. In contrast, the studies on GPCR oligomers by MD are very limited, mainly concerning the self-assembly behavior (Provasi et al., 2015), activation mechanisms (Kim et al., 2017), interaction of dimers (Petersen et al., 2017). Recently, our group probed the effect of the dimerization on the activation and ligand-binding for some GPCRs (Wang et al., 2018a; Zhang et al., 2018b; Zhang et al., 2019). Based on our previous studies on the mechanism of GPCR dimers, we hope to further probe the druggability of their PPI interfaces and its regulation mechanism on the drug function of the receptor, using molecular dynamics simulation, virtual screening and protein structure network. Herein, we selected the CXCR4 dimer (PDBID:3ODU) as a representative of the GPCR dimers, which is sole crystal-structure of the GPCR dimers resolved for the chemokine receptors so far. Ultimately, we screened one ligand, which could significantly enhance the dimer interaction, and revealed its regulation mechanism on the drug binding for the orthosteric site. The observations could provide valuable information for the development of the GPCR PPI drugs.

## Materials and Methods

### Workflow

**Figure 1:**
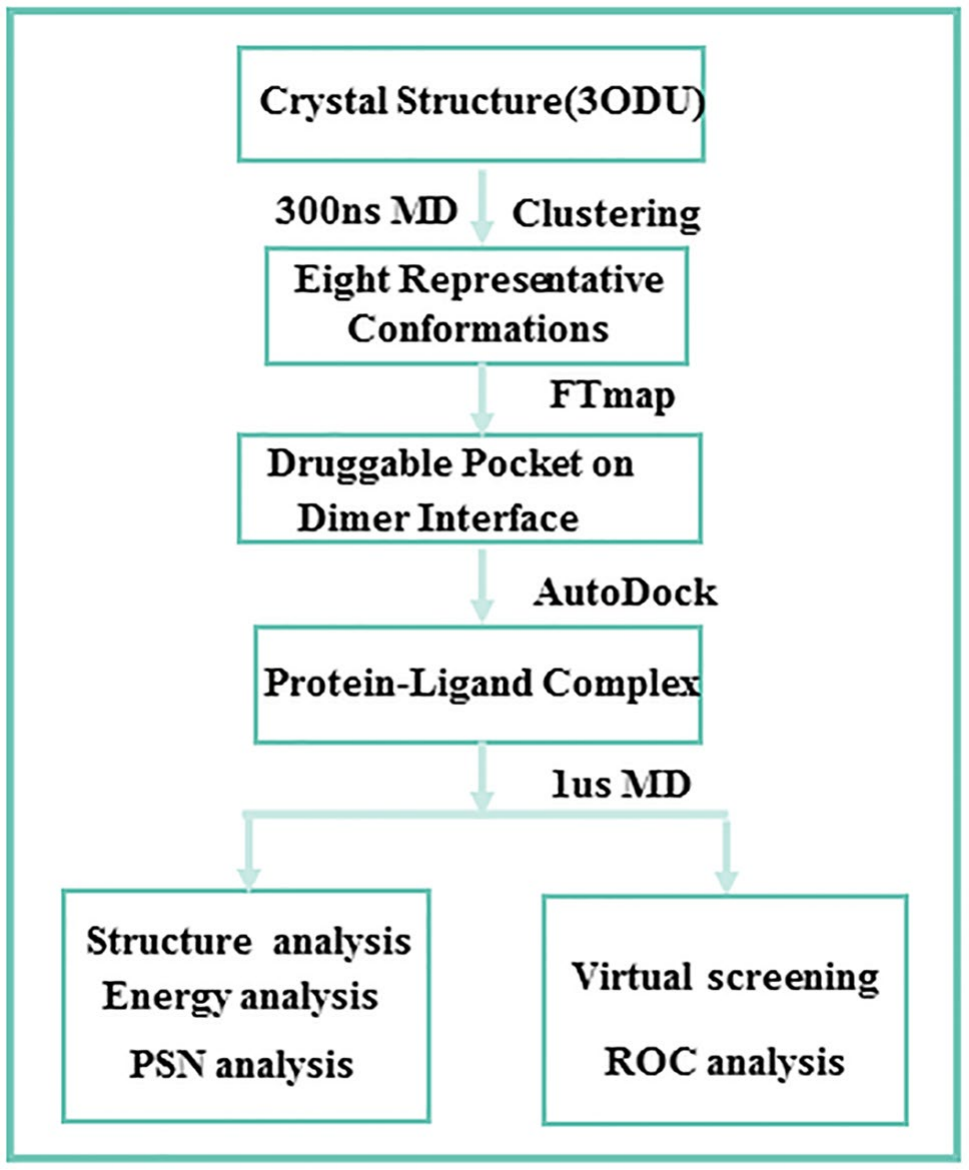
Computational workflow.

**Figure 1** shows the entire workflow. Considering the protein flexibility, 300 ns MD is first carried out for the crystal structure of CXCR4 homo-dimer. Then, according to the root-mean-square-deviation (RMSD) of residues of the dimer interface, eight representative conformations are obtained through clustering. The druggable pocket of the dimer interface is identified by FTmap. Based on the pocket, ligands targeting the PPI interface are screened, and then 1us MD simulations are performed for the four representative complexes of the dimer bound by the PPI ligand. Finally, we discussed the effect of the ligand on the dimerization and the screening performance of the orthostatic site to antagonists, and revealed its regulation mechanism.

### System Preparation

X-ray crystal structure of CXCR4 dimer with a resolution of 2.5 Å was obtained from PDB bank (PDBID: 3 ODU) (Wu et al., 2010). We removed ligands and other non-essential components used for crystallizing and purification, including T4 lysozyme (T4L) inserted between transmembrane (TM) helices V and VI at the cytoplasmic side of the receptor, small isothiourea derivative (IT1t). In addition, crystal water molecules outside the receptor were also deleted. But the crystal water molecules inside the receptor were retained. All protein residues were set to the standard CHARMM protonation state under physiological pH. The receptor was inserted into a palmitoyl-oleoyl-phosphatidyl-choline (POPC) (Filizola et al., 2006) bilayer. Then, water molecules were added to the system, which was described by the TIP3P model. The whole system was neutralized with 0.15M NaCl by CHARMM-GUI (Lee et al., 2015). According to the tertiary structure information of the protein system, the two subunits were manually added with two disulfide bonds between Cys28^N-term^ and Cys274^7.25^, Cys109^3.25^, and Cys186^5.50^.

### MD Simulation

All molecular dynamics simulations were performed by the sander module of AMBER 16 (Case et al., 2016). The MD trajectories were analyzed using the correlation analysis module of AMBER 16 and VMD, as well as some other specific trajectory analysis softwares. Ff14SB force field (Maier et al., 2015) was used for the receptor and the lipid14 force field (Dickson et al., 2014) was utilized for the POPC lipids. Twenty thousand step energy minimization was performed to eliminate bad contacts in the initial structures. After the minimization, the entire system was heated from 0 K to 310 K within 250 ps, then 5 ns NVT pre-equalization was performed at 310 K temperature. Finally, 300 ns and 1 us simulations were carried out using the NPT ensemble at 300 K and 1 bar for the apo dimer system and the dimer bound the PPI ligand, respectively. The cutoff distance of 10 Å was set for nonbonded interactions and the electrostatic interaction was computed by the particle mesh Ewald (PME) algorithm (Essmann et al., 1995). The SHAKE algorithm (Berendsen et al., 1984) was used to constrain all hydrogen-containing bonds. The time step was 2-fs and trajectories were saved at interval of 10 ps for further analysis.

### Clustering Analysis

For the last 200 ns trajectory of the apo dimer system, clustering was carried out using the k-means algorithm (Han and Zhang, 2009; Li et al., 2014) embedded in the ptraj module of the AmberTools package in terms of RMSD of the backbone atoms of 136 residues of TM5-TM6/TM5-TM6 interface (Wu et al., 2010). Consequently, eight classes were obtained (vide in **Supplement 1**) and the center of each class was selected as a representative conformation for subsequent analysis.

### FTMap Analysis

FTmap analysis (Kozakov et al., 2015) was performed in order to identify the druggable pocket in the dimer interface, using FTMap computational map server. The server probes small molecule binding sites using CSM method (Dennis et al., 2002), which places molecular probes on a protein surface to identify the most favorable binding positions. The eight representative structures from the clustering above were individually computed using this server (www.ftmap.bu.edu). Pymol (Janson et al., 2016) was utilized to inspect visually the results.

### Virtual Screening

A ligand set was constructed by a focused chemical compound collection (iPPI-lib) with a total of 51,232 ligand molecules, which was tuned to target PPIs. The PPI-specific database was provided by MTiOpenScreen (Labbé et al., 2015). First, the initial drug-like compounds containing 384,372 PubChem molecules was selected and collected. Then PPI-HitProfiler (Reynès et al., 2010) was used to select PPI-friendly compounds. Finally, these molecules were aggregated by Cluster Molecule Protocol (Accelrys Pipeline Pilot v8.5), resulting in 51,232 drug-like molecules in the final iPPI-lib. Approximately 4,000 molecules (including isomers) were obtained through preliminary screening of MTiOpenScreen, and further docking evaluations were performed using Autodock 4.2 (Morris et al., 2009). All docking input files were prepared by AutoDockTools 1.5.6 (Sanner, 1999) package, and Lattice files for active sites were generated by the AutoGrid 4.2. In order to cover the ligand-binding site, the box site was set to 75 Å × 75 Å × 75 Å with 0.375 Å spacing. The dockings with the flexible ligand and the rigid receptor were performed by AutoDock 4.2. To ensure the accuracy of the result, each ligand was done by 100 docking calculations separately, and 1,000,000 energy evaluations were carried out using Lamarck genetic algorithm for each docking calculation. We selected the docking pose with the lowest binding energy as the best binding mode for further analysis. The ROC (Metz, 1978) plot was used to assess virtual screening performance, which is a curve of true-positive rates versus false-positive rates. They could be calculated in terms of the following equations. 

(1) $$TPR=\frac{TP}{\left(TP+FN\right)}$$

(2) $$FPR=\frac{FP}{\left(FP+TN\right)}$$

Where *TP* (true positive) and *FN* (false negative) refer to the number of active substances in positive and negative classes, respectively. *FP* (false positive) and *TN* (true negative) refer to the number of decoys in positive and negative classes, respectively. The AUC is the area under the receiver operating characteristic curve (Hanley and McNeil, 1982). The larger AUC value, the better the performance of the receptor in screening the active molecules from the decoys. For example, when the AUC value is 0.5, it represents random screening. When the AUC value is 1, the receptor has the strongest ability to screen the active molecules. The AUC value could reflect the affinity of the receptor to a class of active molecules in the ligand set. Therefore, it has been widely used to characterize the performance of virtual screening.

### MMPBSA

As accepted, molecular Mechanics Poisson-Boltzmann surface area (MM/PBSA) (Sun et al., 2014; Sun et al., 2018; Wang et al., 2019; Weng et al., 2019) is a versatile method to calculate the binding free energy Δ*G*_binding_ between two molecules in terms of equation (3).

(3) $$G_{\text{binding}}=G_{\text{complex}}-\left(G_{\text{receptor}}+G_{\text{ligand}}\right)$$

Herein, *G*_complex_, *G*_receptor_ and *G*_ligand_ denote the free energies of the complex, receptor, and ligand, respectively, which could be calculated by MMPBSA.py.MPI algorithm (Miller III et al., 2012) of the SANDER module [vide equations (4)–(6)].

(4) $$G=E_{\text{gas}}+G_{\text{sol}}-TS$$

(5) $$E_{\text{gas}}=E_{\text{int}}+E_{\text{ele}}+E_{\text{vdw}}$$

(6) $$\;G_{sol}=G_{\text{psolv}}+G_{\text{npsolv}}$$

The gas phase energy (*E*_gas_) is calculated by the internal energy (*E*_int_), the electrostatic interaction energy (*E*_ele_) and van der Waals interaction energy (*E*_vdw_) in equation (5). *G*_sol_ denotes the solvation energy, which consists of polar solvation energy (*G*_psolv_) and the nonpolar solvation (*G*_npsolv_) [vide equation (6)]. *G*_psolv_ could be obtained by solving the Poisson-Boltzmann equation while *G*_npsolv_ could be estimated by *γ*×SASA. Herein, *γ* uses 0.0072 kcal Å^-2^ value and SASA denotes the solvent-accessible area of the molecular. The dielectric constants are set to be 1 for the receptor interior and 80 for the external water. *T* represents absolute temperature and *S* is the total conformational entropy. Similar to many computational studies (Niu et al., 2017; Tu et al., 2018), the contribution of entropy is not considered in the calculation of free energy since we mainly concern with the relative change of the binding energy, rather than its absolute value.

### Protein Structure Network

Protein structure network (PSN) (Kannan and Vishveshwara, 1999) could exhibit the structure of proteins as an interaction network. In PSN, residues are served as nodes. If the percentage of interaction [vide equation (7)] between the two nodes is greater than or equal to a given cutoff, the two nodes are connected to one edge.

(7) $$I_{ij}=\frac{n_{ij}}{\sqrt{N_{i}N_{j}}}100\;\;\;\;\;\;\;$$

In equation (7), *I*_ij_ represents the percentage of interaction between nodes *i* and *j*, and *n*_ij_ represents the pair number of side chain atoms within a given distance cut-off range (the default cutoff is 4.5 Å). *N*_i_ and *N*_j_ are the normalization factors of residue *i* and *j*, respectively. Based on the network, we could gain insight into inter-residue communication, which play a vital role for proteins to execute their biological functions. Consequently, PSN has been successfully applied to study unfolding, stability and allosteric interaction (Brinda and Vishveshwara, 2005; Vishveshwara et al., 2009; Gao et al., 2016).

In addition, the shortest paths between pairs of nodes could be obtained through searching PSN by Dijkstra’s algorithm (Dijkstra, 1959), which considers the PSN node inter-connectivities and residue correlated motions. The dynamic cross-correlation (DCC) (McCammon and Harvey, 1988) could be evaluated along an MD trajectory, in which DCC values (*C*_ij_) are computed in terms of equation (8):

(8) $$C_{ij}=\frac{\bar{\left(r_{l}\left(t\right)-\bar{r_{l}}\right)\left(r_{j}\left(t\right)-\bar{r_{j}}\right)}}{\sqrt{\left(r_{i}{\left(t\right)}^{2}-\bar{r_{l}^{2}}\right)\left(r_{j}{\left(t\right)}^{2}-\bar{r_{j}^{2}}\right)}}\;\;\;\;\;\;\;\;\;\;\;$$

*i* and *j* denotes atoms or residues, and *r*_i_(t) and *r*_j_(t) are the corresponding position vectors at time *t*. $\bar{r}$ means the ensemble average over a period time. DCC could characterize the extent of atom or residue movement correlations within a range from 1.0 to -1.0, where 1.0 indicates completely correlated displacements and -1.0 denotes completely anti-correlated displacements. Cross correlation analysis and PSN were performed using Wordom software (Seeber et al., 2011).

## Results and Discussion

### Prediction of the Druggable Pocket in the Interface of the CXCR4 Dimer Based on Representative Conformations

**Figure 2:**
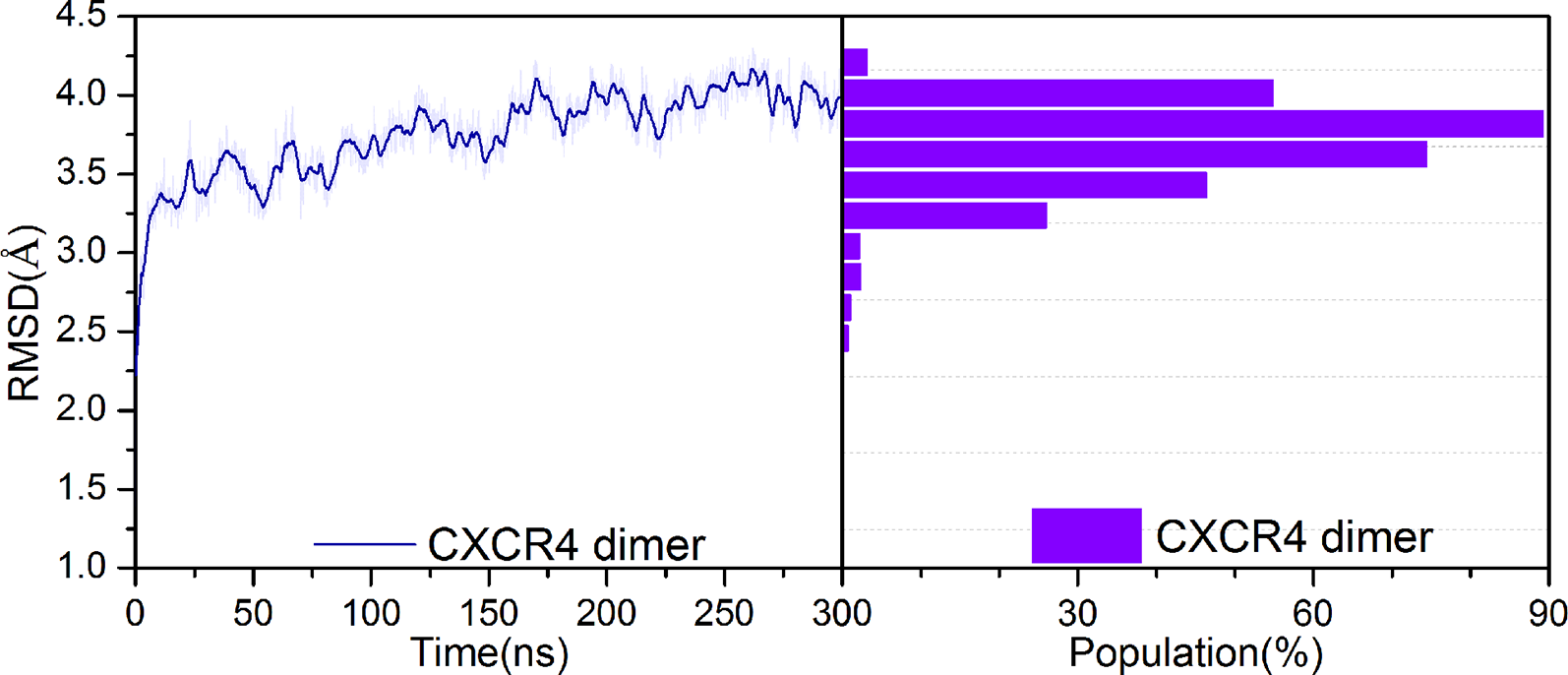
Changes in RMSD values of backbone atoms for the apo CXCR4 dimer along with simulation time (left) and its distribution (right).

**Figure 3:**
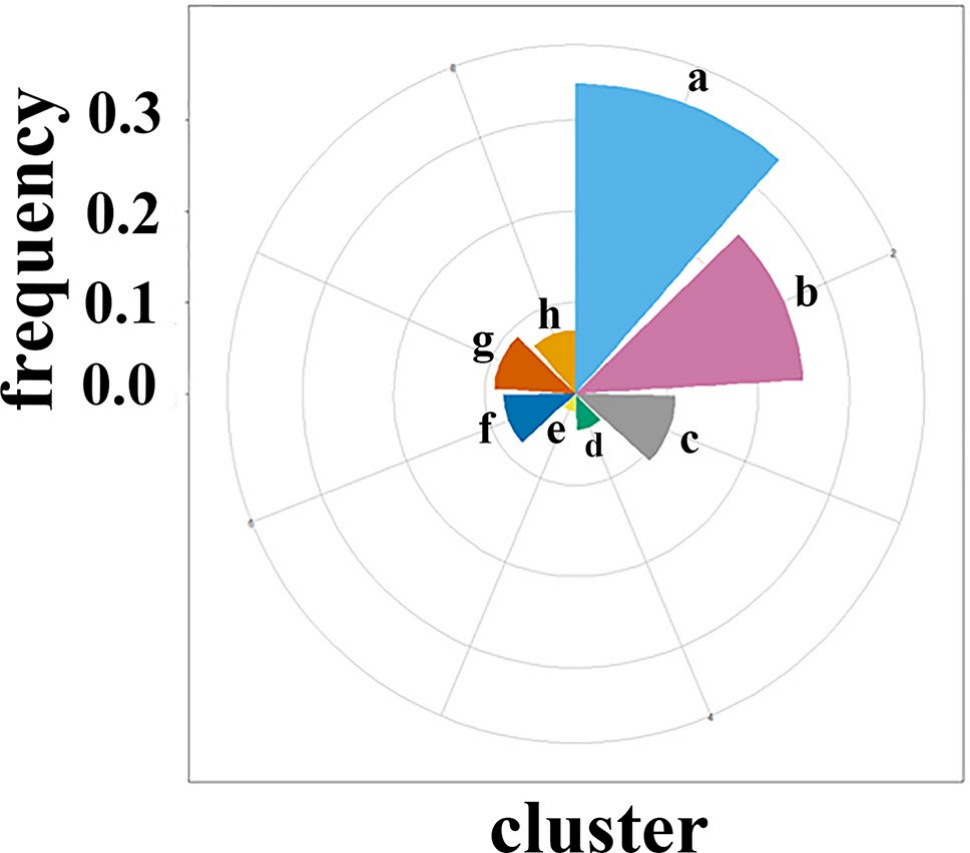
The proportion of the eight representative conformations obtained by clustering in the last 200-ns trajectory for the apo CXCR4 dimer. One druggable pocket was identified by FTmap only for four types of conformations *a*, *b*, *d*, and *g*.

The crystal structure is not completely equal to the functional conformation due to the flexibility of protein, which play a crucial role in the protein function. Thus, we first performed 300 ns MD simulation to obtain representative conformations for the apo dimer. **Figure 2** shows the root-mean-square-deviation (RMSD) of the backbone atoms with respect to its crystal structure for the dimer. It can be seen that the RMSD values present minor fluctuations after 100 ns. Thus, we used the k-means algorithm to cluster the last 200 ns trajectories, based on RMSD of the backbone atoms of 136 residues of the dimer interface (Wu et al., 2010). Consequently, eight classes of the conformations were obtained, as shown in **Supplement 1**. **Figure 3** shows the proportion of conformations for each class and populations of the *a* and *b* classes are significantly higher than the other classes. The center of each class was selected as representative conformation to probe its druggability with the aid of FTmap method. Only one druggable pocket was identified in the dimer interface for the classes *a*, *b*, *d*, and *g*, which account for 72% conformations of the last 200 ns trajectories, thus being highly representative. Furthermore, the druggable pockets in the dimer interfaces are highly similar for the four classes, which are mainly involved in Trp195^5.34^, Val198^5.37^, Phe199^5.38^ of the subunit A, Val197^5.36^, Gln200^5.39^, Phe201^5.40^, Ile259^6.55^, Ser260^6.56^, Ser263^6.59^, and Leu267^6.63^ of subunit B. It was revealed from the CXCR4 crystal structure that the residue Trp195^5.34^, Val197^5.36^, Val198^5.37^, Phe201^5.40^, and Leu267^6.63^ play an important role in the dimerization of dimer (Wu et al., 2010). Thus, it can be assumed that a ligand targeting the pocket could significantly influence the dimerization of CXCR4.

### Screening Potential Ligands to the Druggable Pocket in the Dimer Interface

**Figure 4:**
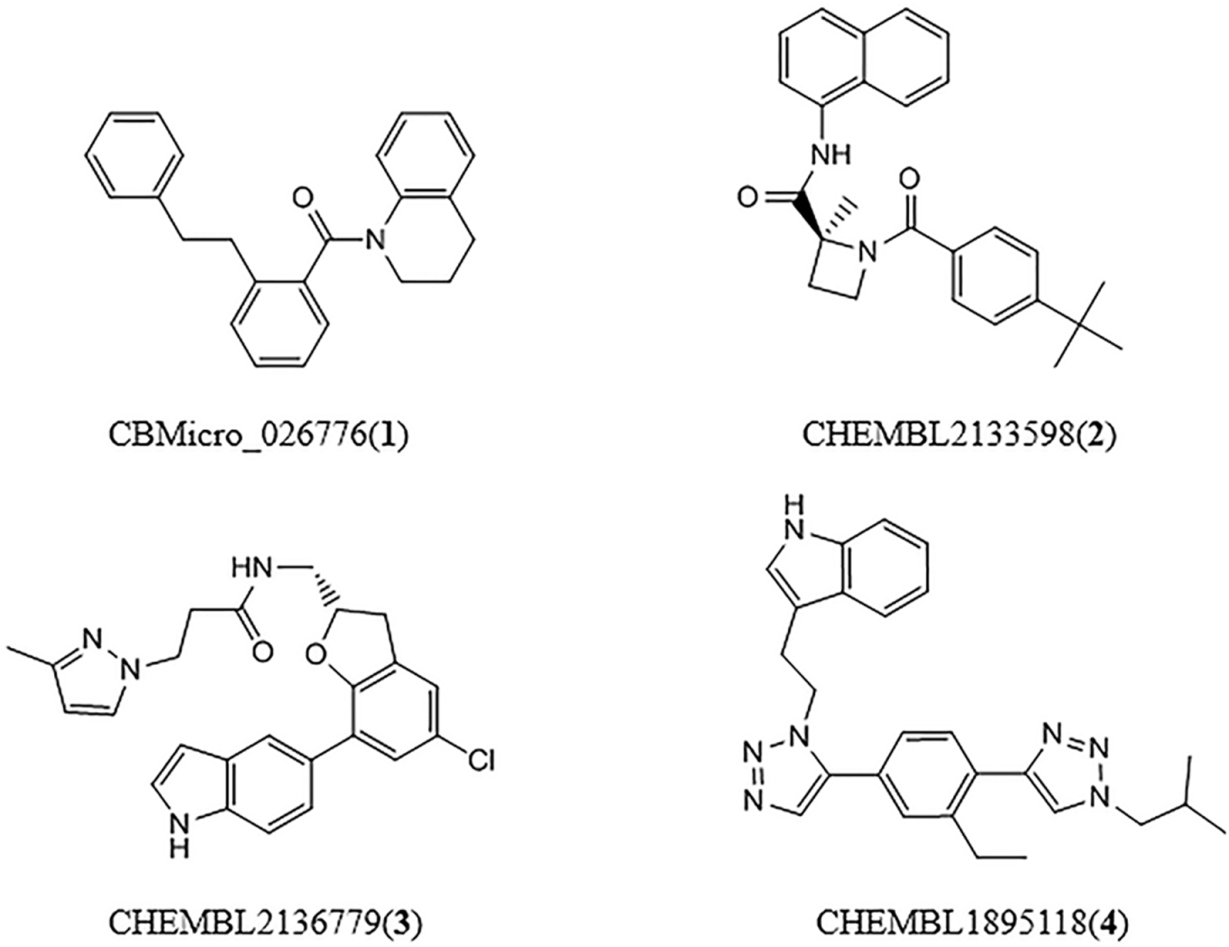
Chemical structural formulas of four ligands (ligand 1 binds to conformational *a*, ligand 2 binds to conformational *b*, ligand 3 binds to conformational *d*, ligand 4 binds to conformational *g*).

The ligand set was constructed by a focused chemical compound collection (iPPI-lib) with a total of 51,232 ligand molecules, which were docked to the four representative conformations of the *a*, *b*, *d*, and *g* classes. The complex with the lowest binding energy was selected as the best binding mode for each of the four classes. Consequently, four small molecules (vide in **Figure 4**) were screened for the four representative conformations, which present best binding. The ligands 1, 2, 3, and 4 correspond to the conformations *a*, *b*, *d*, and *g*, respectively. **Table 1** lists some important physicochemical properties calculated by the SwissADME (Daina et al., 2017) for the four small molecules. It can be seen that their molecular weights are between 340 and 500. LogP values are between 3 and 5. LogS values are between -5 and -6. These properties are in line with those of the PPI drugs reported. Furthermore, the four molecules satisfy “Rule-of-Five” proposed by Lipinski (Lipinski et al., 1997; Lipinski, 2004), which indicates MW≤500, Log P≤5, N or O ≤10, NH or OH≤5, maybe potential drugs.

**Table 1:** Properties of the four ligands targeting the PPI interface.

Ligands	MW (g/mol)^a^	LogP*^b^*	LogS*^c^*	TPSA(Å^2^)*^d^*	Lipinski*^e^*
CBMicro_026776(1)	341.45	4.82	–5.64	20.31	Yes
CHEMBL2133598(2)	400.51	4.64	–5.82	49.41	
CHEMBL2136779(3)	434.92	3.90	–5.05	71.94	
CHEMBL1895118(4)	439.56	4.43	–5.66	77.21	

a Molecular weight.

b Lipid-water partition coefficient.

c Aqueous solubility.

d Topological polar surface area.

e Rule-of-five.

### Effect of the Four Ligands on the CXCR4 Dimerization

**Figure 5:**
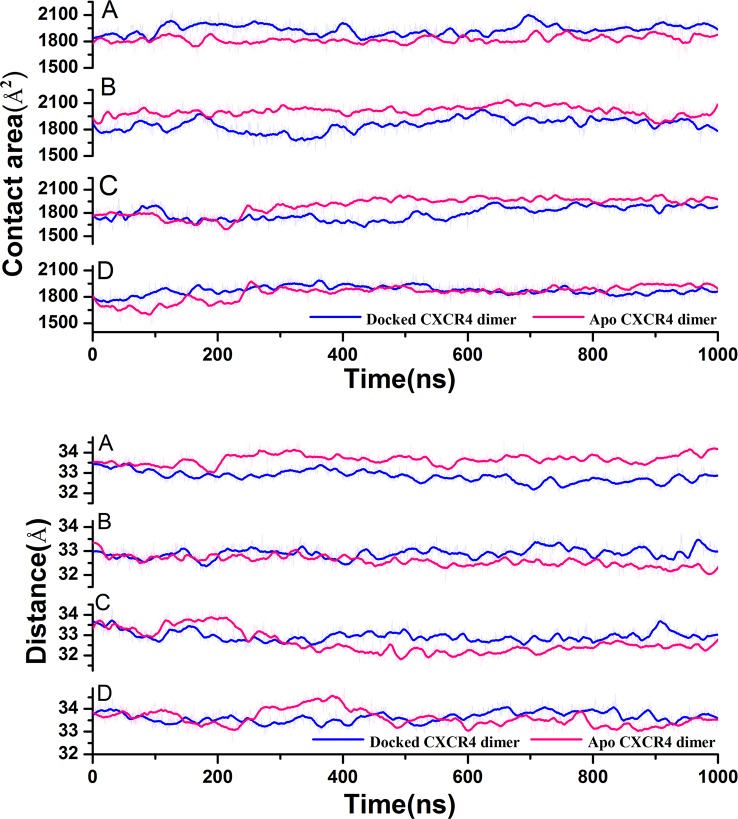
Variations of the contact area and the centroid distance between the two protomers along with simulation time for the CXCR4 dimer with and without the ligand targeting the PPI interface **(A)** Corresponds to conformation *a*, **(B)** Corresponds to conformation *b*, **(C)** Corresponds to conformation *d*, **(D)** corresponds to conformation *g*).

In order to probe the impact of the four ligands on the dimerization of CXCR4, we further performed 1us MD simulation for the four dimer conformations, the interfaces of which were docked by the individual ligand. The centroid distance and the contact area between the two subunits of the CXCR4 dimer were calculated based on the 1 us trajectory, as shown in **Figure 5**. It can be seen from **Figure 5** that only the ligand 1 targeting the conformation *a* reduces the centroid distance between the two subunits and increases their contact area, suggesting enhanced dimerization. However, an opposite trend is presented for the conformations *b* and *d*. For the conformation *g*, the two parameters change little. The observations indicate that the ligand 1 could enhance the dimerization of CXCR4 while the ligands 2 and 3 disfavor the dimerization. The ligand 4 only plays a negligible role in the dimerization. Since our objective is to search the PPI ligand enhancing the dimerization, we only focused on the ligand 1 in the following analysis.

### Interaction Energy Between the Ligand 1 and the CXCR4 Dimer

In order to estimate the interaction strength between the dimer and the ligand, the binding free energy between them was calculated using the MM-PBSA method, based on the last 100 ns trajectory, as shown in **Table 2**. The Δ*G*_binding_ value is -46.77 kcal/mol and van der Waals interaction is main driving force, as judged from -46.18 kcal/mol of Δ*E*_vdw_. In the other words, van der Waals interaction devotes main contributions for the interaction between the CXCR4 dimer and the ligand 1.

**Table 2:** The binding free energy (in kcal mol^-1^) between the CXCR4 dimer and the ligand 1.

Components	Energy (kcal/mol)
Δ*E*_vdw_ *^a^*	–46.18 ± 2.86
Δ*E*_ele_ *^b^*	–3.28 ± 1.20
Δ*E*_int_ *^c^*	0.00 ± 0.00
Δ*E*_gas_ *^d^*	–49.46 ± 3.15
Δ*G*_npsolv_ *^e^*	–4.38 ± 0.20
Δ*G*_psolv_ *^f^*	7.07 ± 1.11
Δ*G*_solv_ *^g^*	2.69 ± 1.08
Δ*G*_binding_ *^h^*	–46.77 ± 2.82

a Non-bonded van der walls contribution from MM force field.

b Non-bonded electrostatic energy as calculated by the MM force field.

c Internal energy arising from bond, angle, and dihedral terms in the MM force field.

d Total gas phase energy.

e Nonpolar contribution to the solvation free energy.

f Polar contribution to the solvation free energy calculated.

g Solvation free energy.

h Binding energy.

*ΔE* *_gas_* *= ΔE* *_ele_* *+ ΔE* *_vdw_* *+ ΔE* *_int_* *, ΔG* *_solv_* *= ΔG* *_npsolv_* *+ ΔG* *_psolv_* *, ΔG* *_binding_* *= ΔE* *_gas_* *+ ΔG* *_solv_*

**Figure 6:**
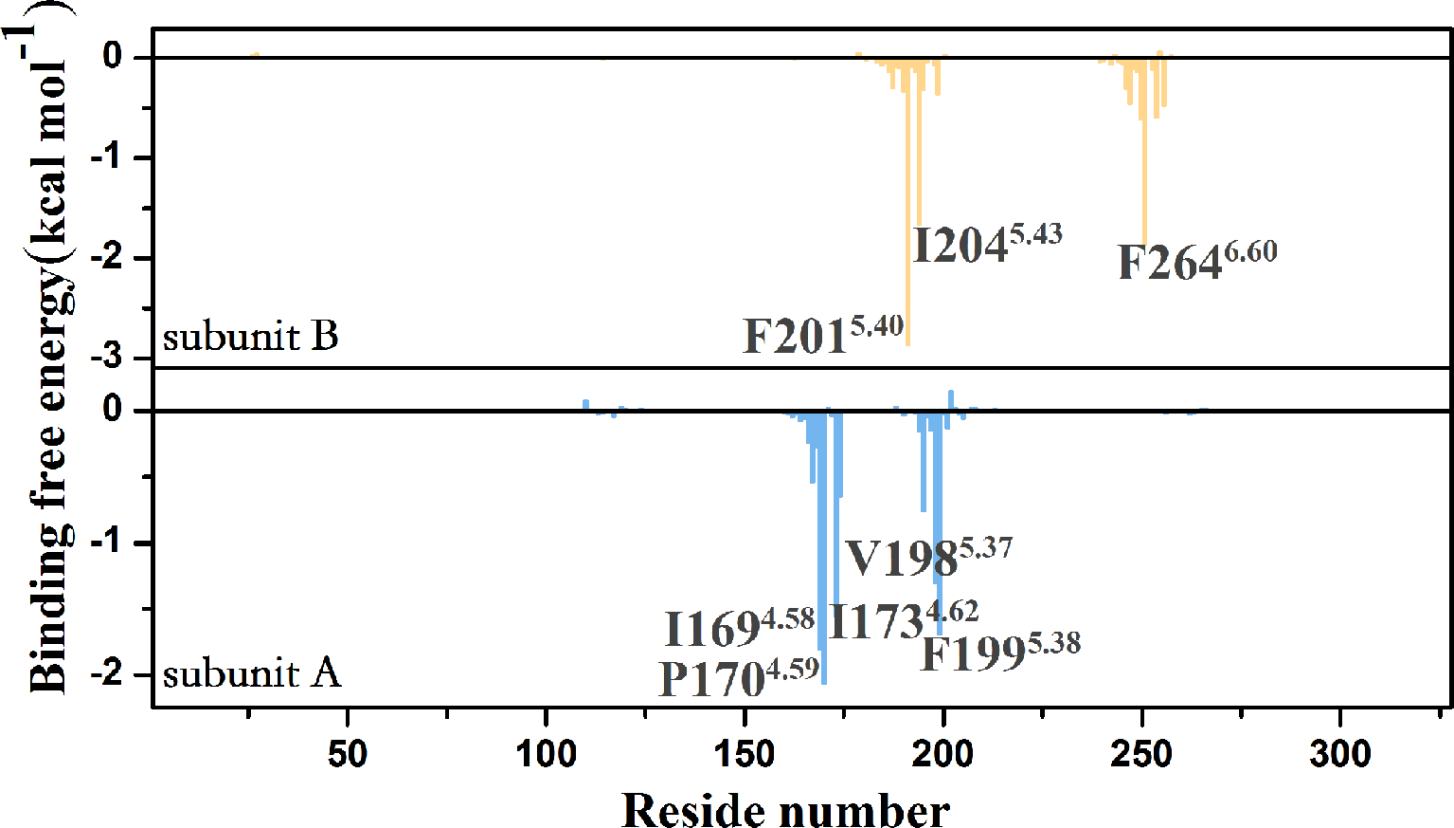
Per-residue decomposition of the binding free energy for the CXCR4 dimer.

**Figure 7:**
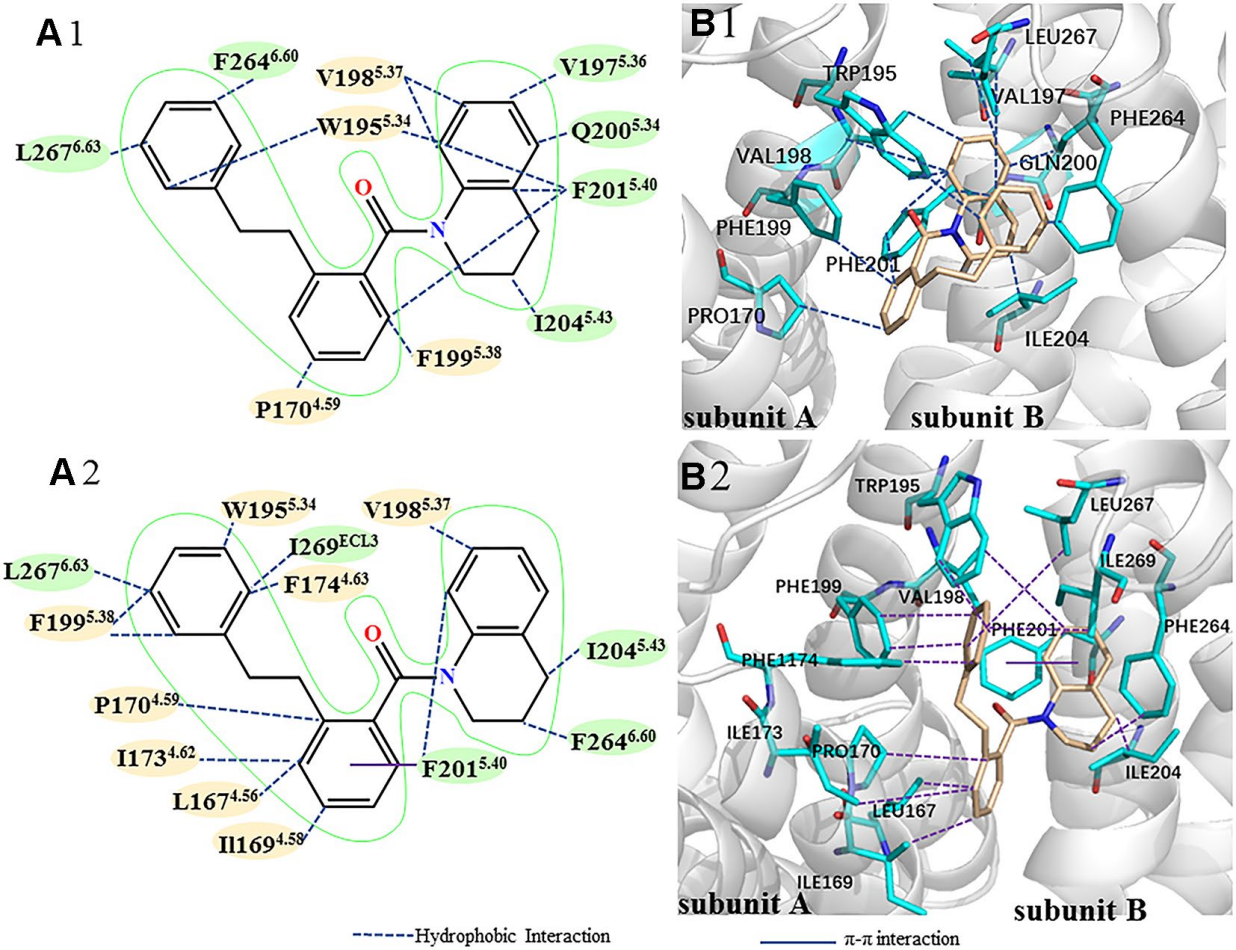
The comparison of binding modes between the CXCR4 dimer and the PPI ligand before (Top) and after (Bottom) 1us simulation. **(A)** 2D diagram of binding mode between the CXCR4 dimer and the ligand (yellow represents residues from the subunit A, green represents residues from the subunit B). **(B)** 3D diagram of binding mode between the CXCR4 dimer and the ligand, blue for residues, orange for the ligand. Hydrophobic and π-π interactions are represented by different lines.

To identify important residues contributed to the ligand binding, we decomposed the binding free energy into the corresponding residue. **Figure 6** shows residues with binding energy less than -1 kcal mol^-1^, including residues Phe201^5.40^, Ile204^5.43^, and Phe264^6.60^of the subunit A, residues Ile169^4.58^, Pro170^4.59^, Ile173^4.62^, Val198^5.37^, and Phe199^5.38^of the subunit B. To identify important groups of the ligand contributed to the binding, we also calculated the interaction between the CXCR4 dimer and the ligand using protein–ligand interaction analysis software (PLIP) (Salentin et al., 2015). **Figure 7** shows the interaction mode between the CXCR4 dimer and the ligand 1 before the simulation and after that. Herein, the snapshot of the lowest energy in the last 100 ns MD trajectory was selected as representative conformation for calculating the binding mode after the simulation. It can be seen from **Figure 7** that the benzene ring of the small molecule devotes main contribution to the hydrophobic interaction between the ligand and the dimer, indicating the importance of the benzene group of the ligand in enhancing PPI. A comparison of the interaction modes in **Figure 7** indicates that TM5 mainly contributes to the binding before the simulation while TM4 also devotes to the binding besides TM5 after 1us simulation. Thus, it should be the interaction between TM4 and the ligand that drives the two subunits closer.

### Effect of Ligand 1 on Drug Screening of Orthosteric Site

**Figure 8:**
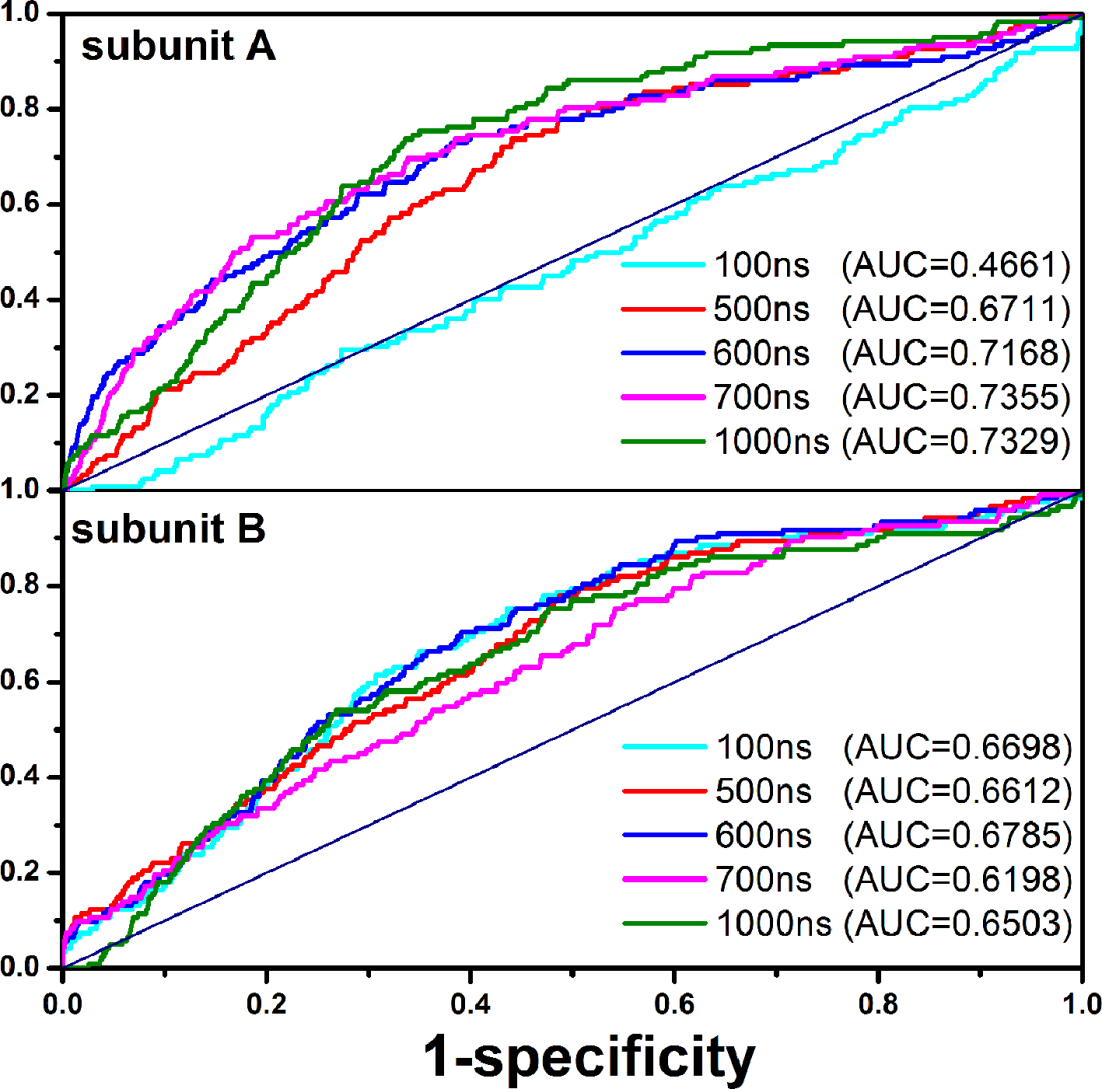
Effects of the PPI ligand on the screening performance of the orthosteric site to the antagonists for the subunit A (top) and the subunit B (bottom).

Since the existing drugs targeting CXCR4 are mainly antagonists, we, herein, focused the impact of the PPI ligand on the selectivity of the orthosteric site to the antagonists. One ligand set was constructed. The active molecules are extracted from the ZINC database (Irwin et al., 2012), GPCR-ligand database (Okuno et al., 2007) and PubChem database (Kim et al., 2015). The decoys stem from the DUD-E database (Mysinger et al., 2012). The ratio of decoys to the active molecules (N activity/N decoy) is 1:36. Consequently, the ligand set contains 1,480 small molecules (40 antagonists and 1,440 decoys). The ligand set was docked to the orthosteric site of the receptor (Wu et al., 2010; Venkatakrishnan et al., 2013; Qin et al., 2015), which consists of Lys25^1.19^, Cys28^1.22^, Arg30^1.24^, Asp97^2.64^, His113^3.29^, Asp171^4.60^, Cys186^5.25^, Asp187^5.26^, Asp262^6.58^, Glu277^7.28^, His281^7.32^, and Glu288^7.32^. **Figure 8** shows the receiver operating characteristic (ROC) curve and the area (AUC) under the ROC.

**Figure 9:**
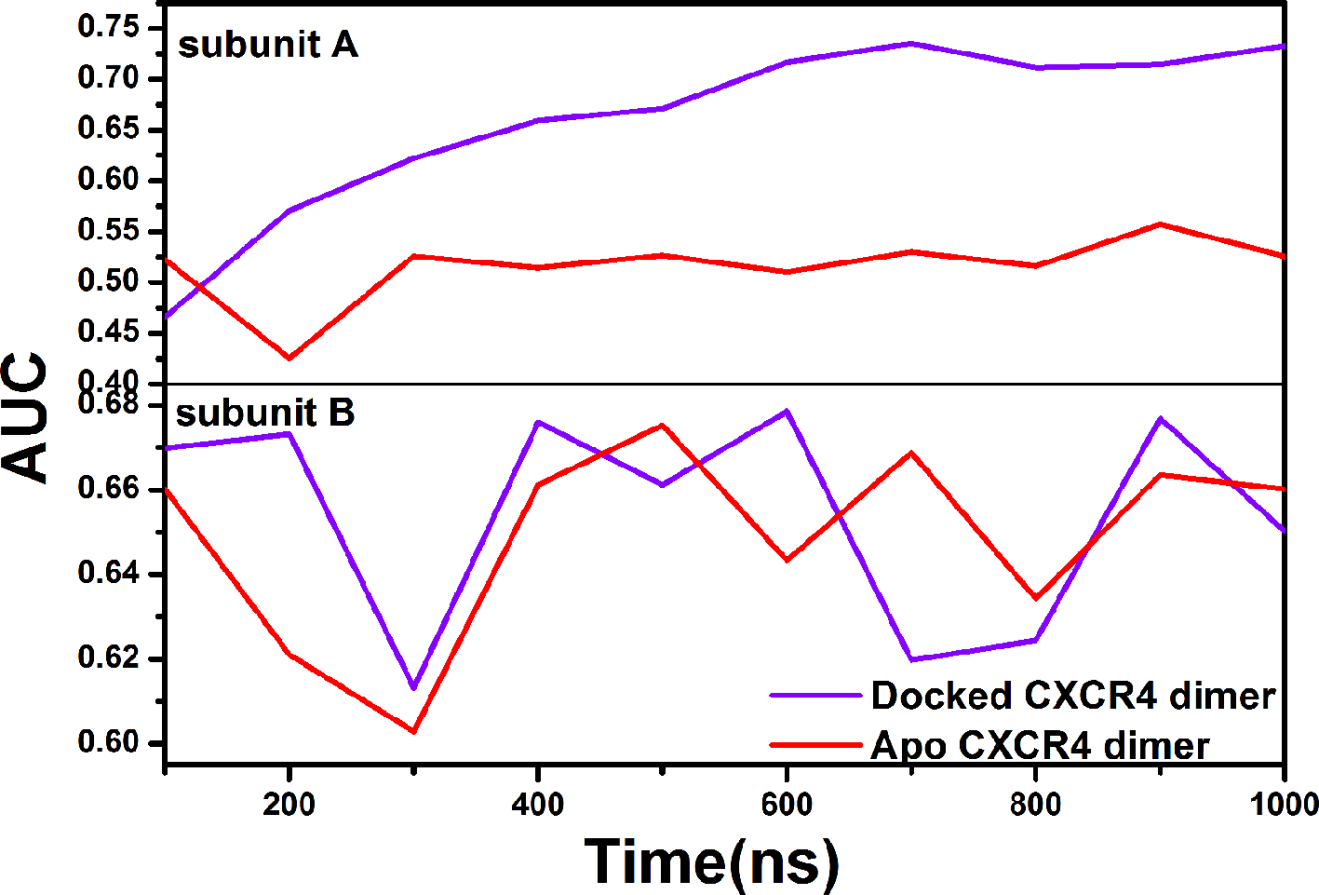
Comparison of the screening performance for the orthosteric sites of the subunit A and the subunit B between the apo CXCR4 dimer (red lines) and the CXCR4 dimer bound by the PPI ligand (purple lines).

It can be seen that the screening performance of the subunit A increases with the simulation time (AUC=0.4661 at 100 ns, AUC=0.6711 at 500 ns, and AUC=0.7329 at 1,000 ns). However, there is little change for the subunit B (AUC=0.6698 at 100 ns, AUC=0.6612 at 500 ns, and AUC=0.6503 at 1 us). In addition, we also compared the drug screening performance of the orthosteric site between the CXCR4 dimer bound the PPI ligand and one without the ligand, as shown in **Figure 9**. Similarly, the PPI ligand improves the screening performance of the subunit A but nearly has no effect on the subunit B, exhibiting asymmetric regulation. The asymmetric effect was also observed for the activation and the ligand binding for some GPCR dimers (Han et al., 2009; Liu et al., 2017).

**Figure 10:**
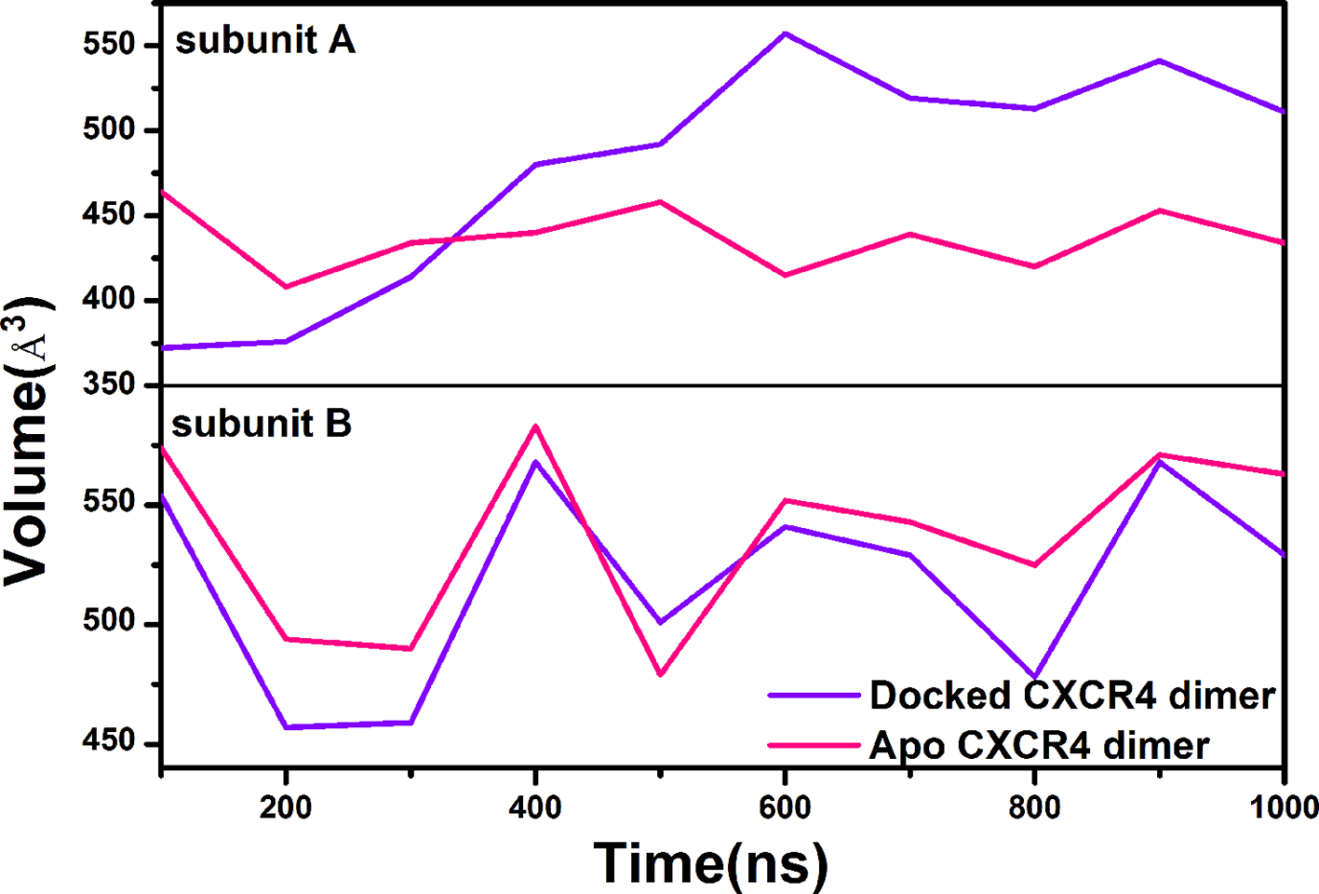
Comparison of volumes for the orthosteric pockets of the subunit A and the subunit B between the apo CXCR4 dimer (red lines) and the CXCR4 dimer bound by the PPI ligand (purple lines).

In order to probe the origin of the asymmetric impact of the PPI molecule on the ligand binding of the orthosteric site for the two subunits, we calculated the pocket volumes of the orthosteric sites of CXCR4, as shown in **Figure 10**. It is clear that the PPI ligand significantly increases the volume of the orthosteric pocket for the subunit A but plays a minor role in the subunit B, which should contribute to the asymmetric screening.

### The Allosteric Pathway for the Regulation Impact of the PPI Molecule on the Ligand Binding of the Orthosteric Site

**Figure 11:**
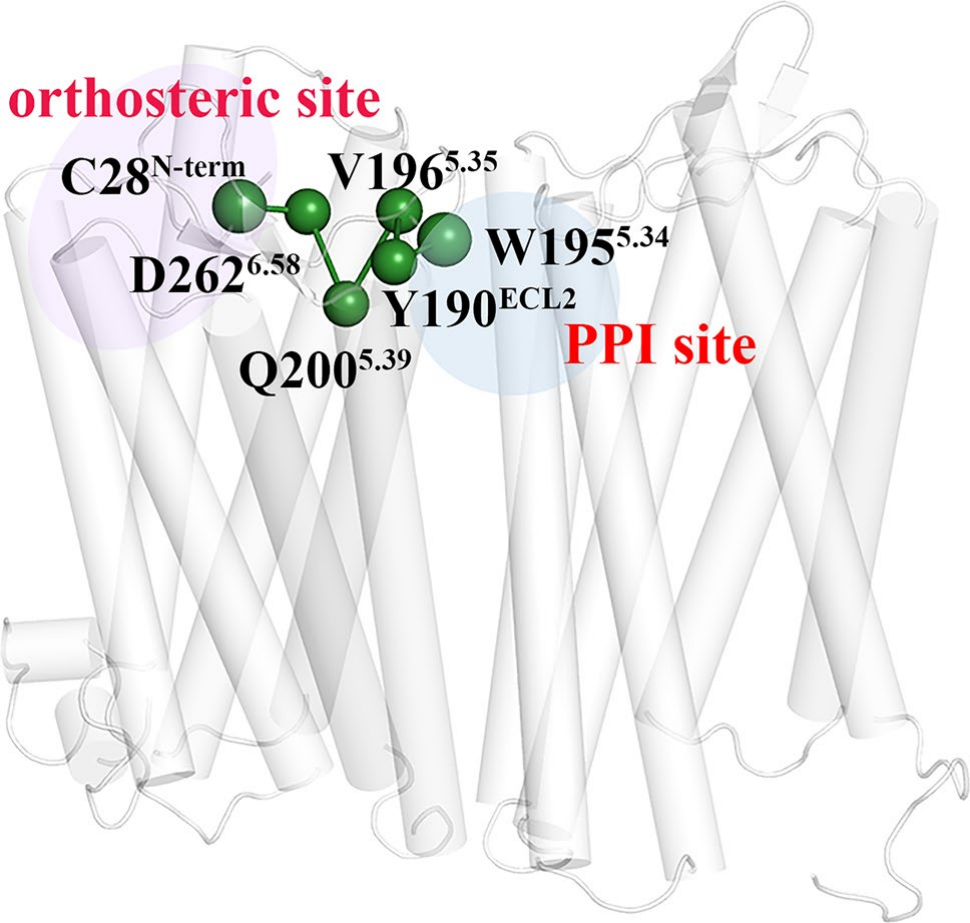
The shortest pathway with the highest frequency between the druggable pocket on the dimer interface and the orthosteric site of subunit A. The blue region represents the PPI pocket. The purple region represents the orthosteric site of the subunit A. The green balls represent the residues composed of the pathway.

In order to probe how the PPI molecule regulates the ligand binding of the orthosteric site of the receptor, we used the protein structure network to identify the allosteric pathway between the druggable pocket in the dimer interface and the orthosteric site of the two subunits. The residues consisted of the two types of the binding pockets are served as the starting and ending nodes in the PSN calculation, respectively, based on the last 100 ns equilibrium trajectory. **Table 3** lists the number of main pathways with frequency higher than 30%. Compared to the dimer without the PPI small molecule, the binding of the PPI molecule significantly increases the number of the pathway for the subunit A while there is little change for the subunit B. The observation suggests that the PPI ligand enhances the role of the interface in regulating the orthosteric site of the subunit A but only plays minor role for the subunit B. As a result, the volume of the orthosteric pocket is increased for the subunit A while the slight change is observed for that of the subunit B. In order to identify important residues in the allosteric regulation pathway, we searched the shortest pathway with the highest frequency between the PPI pocket and the orthosteric pocket for the subunit A. It can be seen from **Figure 11** that the pathway is composed of Trp195^5.34^–Tyr190^ECL2^–Val196^5.35^–Gln200^5.39^–Asp262^6.58^–Cys28^N-term^. As revealed above, Trp195^5.34^ is an important residue contributed to the binding of the PPI ligand. Residue Tyr190^ECL2^ locates in ECL2, which was revealed to be switch for the ligand binding in the orthosteric site (Scarselli et al., 2007; Arkin et al., 2014). Residue Gln200^5.39^ plays a specific role in the dimerization of CXCR4 dimer (Altwaijry et al., 2017). The residues Cys274^7.25^ and Cys28^N-term^ bind closely through disulfide bonds, which play an important role in the formation of entrance to the ligand binding pocket at orthosteric site (Wu et al., 2010; Pawig et al., 2015). Residue Asp262^6.58^ is an important residue for the binding of orthosteric site ligands (Wu et al., 2010; Qin et al., 2015). It can be seen that most of the residues composed of the allosteric pathway are associated with the ligand binding, which should be the reason why the PPI ligand significantly affect the ligand binding pockets, in turn influence its screening performance to the ligands. Although there is no report on the importance of the residue Val196^5.35^ of this pathway, our observations suggest that the residue Val196^5.35^ is also important for the ligand binding of the dimer and should be concerned by experiments. In addition, we also searched the shortest pathway with the highest frequency between the PPI pocket and the orthosteric pocket of the subunit B, as shown in **Supplement 2**. The pathway is composed of Ile269^ECL3^–Phe264^6.60^–Ile270^ECL3^–Ile265^6.61^–Glu277^7.28^, only Glu277^7.28^ of which was reported to be the pocket residue of the orthosteric site (Wu et al., 2010; Venkatakrishnan et al., 2013; Qin et al., 2015). Compared to the pathway of the subunit A, there are fewer residues involved in the ligand binding for that of the subunit B, which should contribute to the observation above that the PPI ligand plays a minor role in influencing the screening ability of the subunit B to the antagonists.

**Table 3:** The number of communication pathways between the binding pocket of the dimer interface and the orthosteric binding pocket (frequency above 30%), derived from the last 100 ns trajectory of the 1 us simulation.

	*^a^*Docked-dimer	*^b^*Free-dimer
Subunit A	78	56
Subunit B	79	77

a The dimer docked by the PPI ligand.

b The apo dimer.

## Conclusions

PPIs offer a rich source of novel drug targets. As the largest family of drug-targeted proteins, it was evidenced that GPCRs could form the dimers through the protein–protein interaction. Unfortunately, the drugs targeting the PPI interface of the GPCR dimers have not been explored so far. In the work, we utilized molecular dynamics simulation coupled with the virtual screening and the protein structure network to probe the druggability in the PPI interface of CXCR4 homodimer and its regulation mechanism on the receptor structure and the drug screening ability of the orthosteric site.

One druggable pocket is identified in the PPI interface. One small molecule is screened from the PPI drug-like small molecule dataset which could enhance the dimerization mainly through hydrophobic interactions between the benzene rings of the PPI molecule and TM4 of the receptor. The enhancement of PPI by the small molecule changes the screening performance of the two subunits to the antagonists targeting the orthosteric pocket. One subunit exhibits an enhanced screening performance to the antagonists while the minor change is observed for the other subunit, exhibiting an asymmetric cooperativity. The structural analysis indicates that the negative cooperativity should be attributed to the asymmetric change in the orthosteric pocket volumes induced by the binding of the PPI molecule, which leads to the significant increase in the pocket volume of the subunit A but only plays a minor role for the subunit B.

The results of PSN reveal that the number of the regulatory pathways from the PPI pocket to the orthosteric pocket is significantly increased for the subunit A while a minor change is observed for the subunit B, which should contribute to the asymmetric change of the binding pockets between the two subunits. In addition, one main regulatory pathway from the PPI binding site to the pocket of the subunit A is identified, revealing that the PPI ligand molecule allosterically regulates the structural change of the orthosteric pocket of the subunit A mainly through the pathway consisted of Trp195^5.34^–Tyr190^ECL2^–Val196^5.35^–Gln200^5.39^–Asp262^6.58^–Cys28^N-term^. These residues were revealed to significantly contribute to the dimerization and the ligand binding to the PPI interface and the orthosteric site. Consequently, the PPI small molecule could significantly regulate the dimerization and the screening ability of the orthosteric site to the ligands.

It is first time revealed the druggability of the GPCR dimer interface and its role in influencing the drug recognition ability of the orthosteric site. Since the antagonists of CXCR4 are used to treat CXCR4-related diseases like AIDS and some cancers, it is reasonable to assume that the PPI molecule identified from the work should enhance their drug efficacies. In addition, the strategy proposed by the work could be applied to probe the other GPCR PPI drugs.

## Data Availability Statement

All datasets generated for this study are included in the article/**Supplementary Material**.

## Author Contributions

XP designed the experiments. YY and YG finished all the molecular dynamic simulations and analyzed the trajectories. LS organized the main manuscript text and plotted all the figures. XP, CL, and ML revised the manuscript. All authors contributed to the work.

## Funding

This project is supported by the National Science Foundation of China (Grant No. 21573151), NSAF (Grand No. U1730127), and National Natural Science Youth Foundation of China (Grand No. 2170511).

## Conflict of Interest

The authors declare that the research was conducted in the absence of any commercial or financial relationships that could be construed as a potential conflict of interest.
