# Supplementary material for: Probing the Druggablility on the Interface of the Protein–Protein Interaction and Its Allosteric Regulation Mechanism on the Drug Screening for the CXCR4 Homodimer
Source: Front Pharmacol. 2019 Nov 7;10:1310. doi: 10.3389/fphar.2019.01310 (PMC6855241; doi:10.3389/fphar.2019.01310)
Supplement: Supplementary file 1 [file DataSheet_1.docx]

Supplementary Material


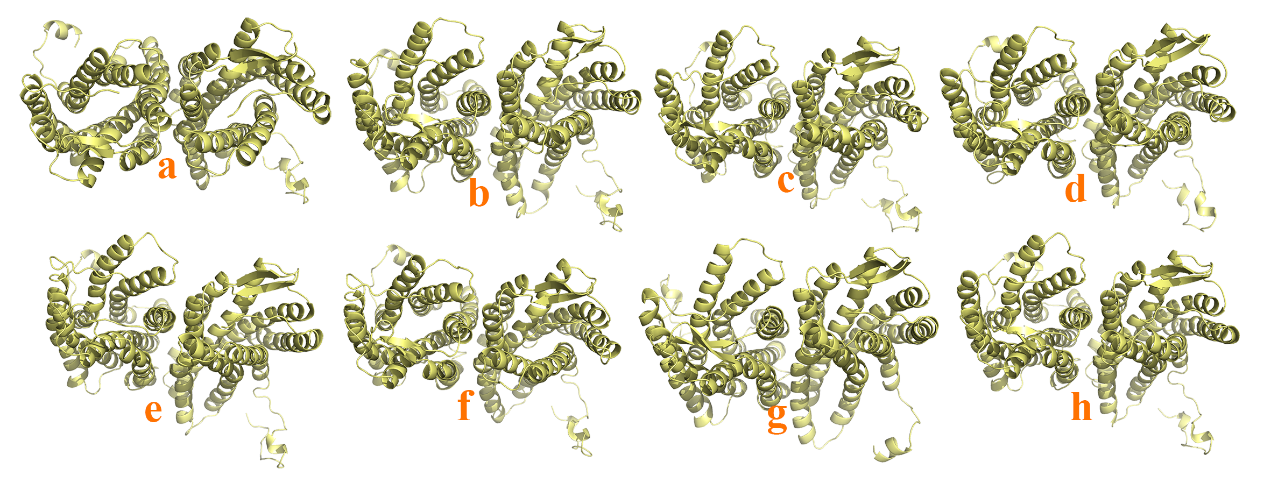


**Supplement 1**. Structures of eight representative conformations, which are obtained by the clustering analysis based on RMSD values (Å) of the backbone atoms of residues of TM5-TM6/TM5-TM6 interface with respect to the crystal structure 3ODU.


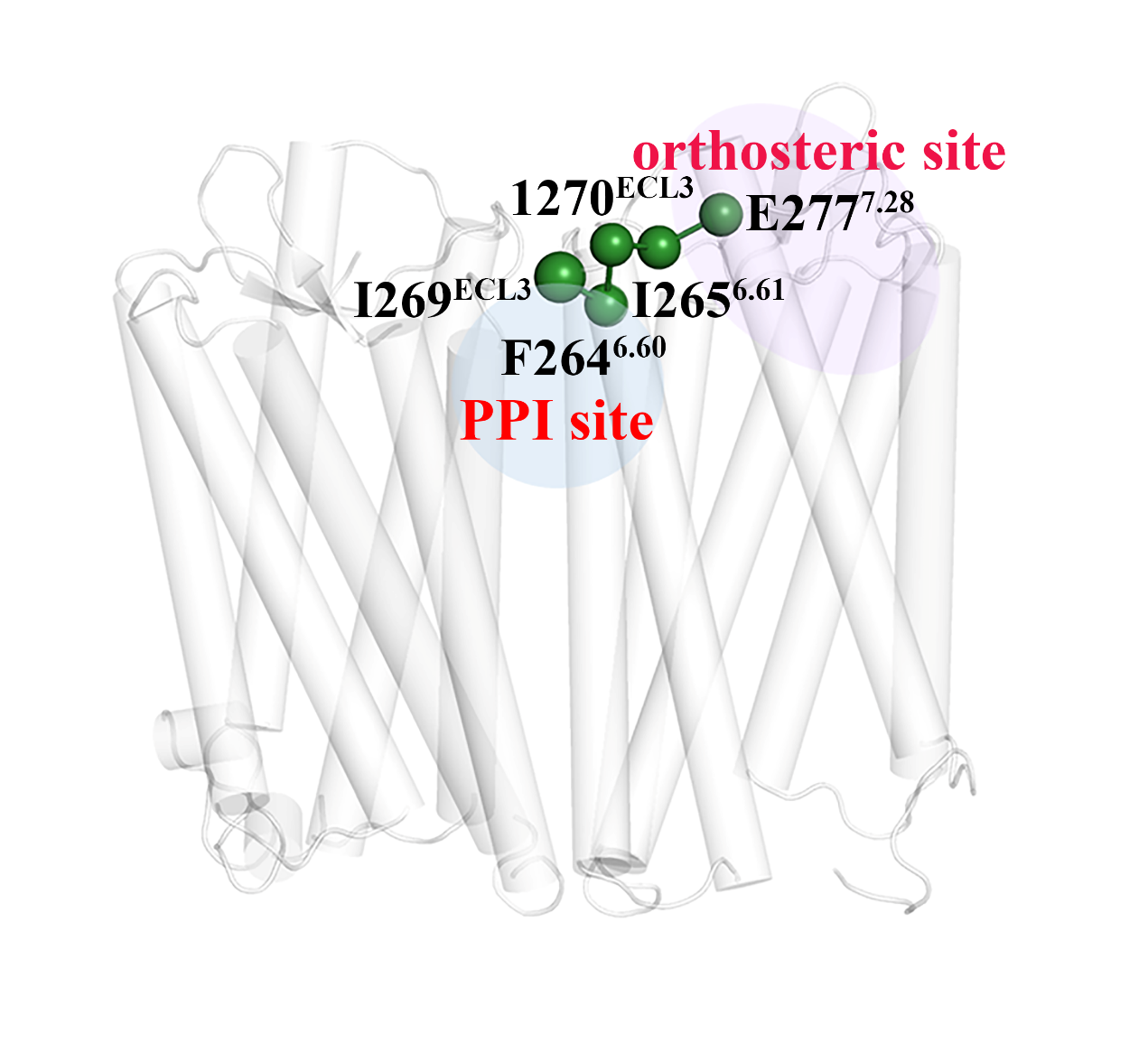


**Supplement 2.** Pathway with the highest frequency between the binding site of CXCR4 dimer interface and the orthosteric site of subunit B.
